# Association between phase angle from bioelectrical impedance and dietary intake in athletes: a cross-sectional study

**DOI:** 10.1017/jns.2025.23

**Published:** 2025-06-10

**Authors:** Christianne de Faria Coelho-Ravagnani, Lorena Cristina Curado Lopes, Allan da Mata Godois, Analiza Mónica Silva, Vitor Cordeiro, Adolfo Henrique Costa dos Santos, João Felipe Mota

**Affiliations:** 1 Graduate Program in Movement Sciences, Research Group in Exercise and Nutrition in Health and Sports Performance – PENSARE, Federal University of Mato Grosso do Sul, Campo Grande, MS, Brazil; 2 University Center of Mineiros, Mineiros, GO, Brazil; 3 University Center of Varzea Grande, Varzea Grande, MT, Brazil; 4 Exercise and Health Laboratory, CIPER, Faculdade de Motricidade Humana, Universidade de Lisboa, Lisbon, Portugal; 5 Departament of Movement Sciences and Sports, Training School of Sport Sciences, The University of Jordan, Amman, Jordan; 6 Faculty of Nutrition, Federal University of Goias, Goiania, GO, Brazil

**Keywords:** Athletes, Bioelectrical impedance, Diet, Phase angle, Proteins, BIA, bioelectrical impedance analysis, FFM, fat-free mass, FFMI, fat-free mass index, PhA, phase angle, R, resistance, Xc, reactance, SD, standard deviation, SPSS, statistical package for social science, 24HR, 24-hour dietary recall interview

## Abstract

Phase Angle (PhA) has emerged as an important parameter to monitor body composition, fluid status, muscle integrity, and physical performance among athletes. However, limited information exists regarding the associations between PhA and dietary intake, especially in athletes. This study aimed to identify the dietary intake components associated with PhA in athletes. This cross-sectional observational study was carried out with 153 athletes across 17 sports. Body composition was assessed by tetrapolar multifrequency BIA, and dietary intake by 24-hour dietary recalls administered on non-consecutive days. Reported foods and supplements were categorised into different groups (i.e. cereals, vegetables, fruits, beans and nuts, meat and eggs, dairy products, oils, and sugars), with portions established based on the food’s total energy content. Fat-free mass and fat-free mass index were higher in male compared to female athletes, potentially influencing PhA (6.6º vs 5.5º; P < 0.01). Multiple linear regression analysis indicated that protein intake was a significant predictor of PhA in athletes. This association remained significant even after adjustments for sex, age, and fat-free mass (R^2^ = 0.48, β = 0.27, P = 0.02). The positive correlation observed between dietary protein and PhA reinforces the need for adequate daily protein intake to enhance PhA in athletes. Further studies investigating the effect of diet-induced changes in PhA within the athletic population are necessary.

## Introduction

Bioelectrical impedance analysis (BIA) is a non-invasive and cost-effective method widely employed for body composition assessment in clinical practices and sports medicine.^(1)^ In the context of body composition and nutrition assessment, some raw bioelectrical impedance parameters, such as resistance (R), reactance (Xc), and phase angle (PhA), have been used for monitoring athletes and other populations.^(1–3)^ While the R emerges from intra- and extracellular body fluids, representing the force that a biological conductor opposes to the electric current, the Xc emerges from the cell membranes, representing their capacitive property, reflecting the cell integrity.^(4)^ The combination of both types of resistance (i.e. R and Xc) is called impedance, which generates the PhA. The PhA is derived from the equation PhA (°) = [−arc tangent (Xc/R) × 180°/π] and serves as an indicator of intra- and extra-cellular fluid distribution, cell mass, and volume.^(2)^

In the realm of sports, PhA has emerged as an useful, safe, non-invasive, and easy to measure tool, particularly in assessing body composition (especially body cell mass),^(3)^ fluid status,^(5,6)^ muscle injuries,^(7)^ and performance^(8,9)^ over training and competitions seasons.^(6,10,11)^ Low PhA values can be observed in athletes from many sports due to fluid imbalance, dehydration, musculoskeletal injuries, muscular glycogen depletion, and lean tissue loss resulting from high frequency and intensity training sessions and competitions.^(10)^ Conversely, PhA is positively associated with muscle mass and strength.^(12)^ In a systematic review aimed at assessing the variability of PhA across different sports, the authors suggested that muscle-strengthening training induces greater increase in PhA compared to endurance training. However, due to the limited number of studies involving athletes, in addition to weaknesses in study designs or small sample sizes, definitive conclusions about these differences are not yet possible.^(3)^

For instance, elite athletes are expected to have a higher PhA compared to their matched non-athletic peers.^(13,14)^ The average PhA in young (19–38 years) and healthy individuals is 7.3^o^ for males and 6.4^o^ for females,^(15)^ whereas, in athletes from different sports, the 50th percentiles range between 7.6^o^ to 7.7^o^ and 6.7^o^ to 6.9^o^ for male and female athletes, respectively.^(16)^ This higher PhA is possibly related to the greater muscle mass and optimal nutrition found in these populations, important characteristics for performance in sports.^(6,17)^ Since nutrition affects morphological (e.g. percentage of body fat and fat-free mass) and physiological parameters (e.g. cell hydration) in response to training, it may also indirectly affect the PhA.

Despite the potential significance of PhA in sports nutrition, there is a lack of comprehensive studies investigating the influence of dietary intake on PhA. In disease conditions^(18)^ and various age groups,^(19,20)^ lower PhA has been associated with diet inadequacies (i.e. insufficient carbohydrate, protein, or energy intake). Interestingly, positive correlations between PhA and meat intake have been observed in patients with diabetes and hypertension^(18)^ and in healthy young people,^(20)^ emphasising the potential impact of the diet composition on PhA. PhA could provide dietitians with a simple way to monitor the effects of dietary strategies (e.g. increasing daily protein ingestion or supplementation strategies) targeting dietary adequacy during the training seasons, and the application of raw parameters of BIA could highlight opportunities for future research in the field of sports nutrition.^(4)^ Nonetheless, to the best of our knowledge, no studies have investigated the influence of dietary intake on PhA in athletes. Thus, the current study aims to determine which dietary intake components are associated with PhA.

## Methods

### Study design and participants

This is a cross-sectional study carried out with a convenience sample of 153 athletes (aged 14–48 years old, BMI 17.6–36.5 kg/m^2^) across 17 sports, including: beach volleyball, volleyball, bodybuilding, football, cycling, tennis, futsal, soccer, Brazilian jiu-jitsu; judo, karate; kung-fu, mixed martial arts, taekwondo, swimming, track and field, and triathlon. Athletes were recruited from sports federations, sports club managers, or indicated by coaches. The inclusion criteria were: 6 or more hours of training/ week and 6 days/ week, enrolled in regional, national, and international level championships. Exclusion criteria were disabled athletes, pacemaker users, and athletes using pins, plates, or other types of metal objects in their body. This study was conducted according to the guidelines laid down in the Declaration of Helsinki, and all procedures involving human subjects/patients were approved by the Human Beings Ethics and Research Committee of the University Hospital Júlio Müller under protocol number 79957217.6.0000.0021 and 25620713.3.0000.5541. Participants or guardians (for athletes under 18 years) were fully informed about all study aspects and signed an informed consent before participating in the investigation. Participants were invited to attend an interview and screening physical test at the Nucleus of Physical Fitness, Informatics, Metabolism, Sports and Health, located at the Federal University of Mato Grosso, Brazil, from 2013 to 2016.

### Body composition

Body weight was measured with a calibrated scale (Welmy 110CH, Brazil), without shoes and wearing light clothes, to the nearest 0.01 kg. Height was measured to the nearest 0.1 cm with a stadiometer (Sanny, São Bernardo do Campo, Brazil).

The PhA, fat-free mass and percentage of body fat were obtained by a tetrapolar (8-Point Tactile Electrode) impedance analyser (Inbody® S10, Cerritos, USA), using an operating frequency of 50 kHz. The equipment provides bioelectrical impedance (Z), Reactance (Xc), and Phase angle at the whole body (which was used in this study) and each of 5 Segments (right arm, left arm, trunk, right leg, and left leg) and does not use empirical estimations based on age, gender, and more to calculate body composition. The InBody S10 features an auto-calibration function upon powering on, without the need for additional calibration. The principle of the InBodyS10 analyser is that the volume of body water is calculated first with a measured impedance value. Then, the fat-free mass (which includes minerals and soft lean mass) is obtained using the volume of body water. Body fat mass is subsequently determined by deducting the fat-free mass from the measured weight. The fat-free mass index (FFMI) is calculated by dividing the fat-free mass in kilograms by the square of the height in metres. The InBodyS10 measures of fat-free mass exhibited excellent precision in athletes (with a coefficient of variation %CV < 1.0%) (Cataldi, 2024).

Participants were instructed to abstain from consuming food and beverages in the previous 4 h, alcoholic and caffeinated drinks for at least 48 h, and strenuous physical exercise for at least 24 h before the test. Anthropometric and BIA assessments were performed on Saturday mornings, specifically between 8 and 9 a.m. Athletes were asked to empty their bladder before the measurement, remove all metal-containing objects and adopt a supine position with limbs slightly spread apart from the body. The touch-type electrodes were attached according to the manufacturer’s recommendations. Electrodes were attached to both hands (to the thumbs and the middle fingers) and feet (positioned between the examinee’s ankle bone and heel).^(21)^

### Dietary intake

Participants reported their food intake for two non-consecutive days (i.e. on Wednesday and Saturday) in a face-to-face 24-hour dietary recall interview (24HR). Athletes were asked to declare all food items, dietary supplements, and drinks consumed in the previous 24 hours. Additionally, details were requested about preparation methods, ingredients used in mixed dishes, and the brand names of commercial products and ready-to-eat foods.^(22,23)^ All foods and beverages were estimated and recorded in standardised household measures (e.g. glasses, cups, spoons).^(22)^ Dietary supplements were recorded according to the manufacturer’s suggested serving size (e.g. scoops, capsules, pills). A photograph series depicting different amounts of a particular food was used to estimate portion sizes.^(24)^ The Brazilian food database was used to calculate the daily energy and nutrient intake from food and nutritional supplements (software Virtual Nutri^®^, São Paulo, SP, Brazil). We accepted the manufacturer’s declaration of the nutritional composition of supplement items^(25,26)^ A Brazilian food guide was used to determine portions of food groups.^(27)^ Foods and supplements reported were categorised into eight different food groups, and the number of portions was established according to the total energy of food items. Food groups and the energy equivalent per portion were: cereals (150 kcal), vegetables (15 kcal), fruits (35 kcal), beans and nuts (55 kcal), meat and eggs (190 kcal), dairy products (120 kcal), and oils and sugars (73 kcal). The criterion used to categorise supplements into food groups was based on the most prevalent nutrient. For instance, whey protein supplements were classified into the ‘dairy products’ group, albumin into the ‘meat and eggs’ group, and maltodextrin into the ‘sugars’ group. Supplements with very low or no calorie supplements, such as vitamins and minerals, were not categorised into food groups.

### Statistical analyses

Statistical analyses were performed using Statistical Package for Social Science (SPSS) software, version 20.0 (IBM SPSS Inc., Chicago, IL, USA). Data are presented as mean ± standard deviation (SD). The Shapiro Wilk test was applied to assess the normality of the distribution of the variables. Subsequently, comparisons between sexes were performed using Student’s *t*-test for parametric variables and the Mann-Whitney *U* test for non-parametric variables.

A stepwise multiple regression analysis was conducted to identify the dietary predictors of phase angle (PhA). This method systematically adds or removes variables based on their statistical significance. The variables considered in the initial regression models included carbohydrate, protein, and lipid intake (g/day), as well as food group consumption (portions/day): vegetables, fruits, beans and nuts, meat and eggs, dairy products, oil, and sugar.

The stepwise procedure selected only the variables that significantly contributed to explaining the variance in PhA for inclusion in the final model. The analysis was performed in five steps: Model 1 represented the unadjusted analysis (crude model); Model 2 was adjusted for age; Model 3 was adjusted for age and sex; Model 4 was further adjusted for age, sex, and fat-free mass; and Model 5 was adjusted for age, sex, fat-free mass, and total energy intake. Statistical significance was set at P < 0.05.

## Results

The sample was composed of 153 athletes (80.4% males), and the characterisation data (age, body composition, and PhA) are presented in Table 1. Male athletes demonstrated significantly higher values in age (Δ = 4.00 years old), weight (Δ = 20.10 kg), height (Δ = 0.16 m), fat-free mass (Δ = 22.10 kg), FFMI (Δ = 4.30 kg/m^2^), and PhA (Δ = 1.10º) compared to their female counterparts. Female athletes had a significantly greater body fat percentage compared to male athletes (Δ = 9.5%; P < 0.05).

**Table 1.** Differences in age, body composition, and bioelectrical impedance phase angle according to sex

	Male athletes (*n* = 123)	Female athletes (*n* = 30)	Total (*n* = 153)
**Age (y)**	22.1 ± 7.3	18.1 ± 4.45^b^	21.3 ± 6.9
Weight (kg)	73.5 ± 11.4	53.4 ± 6.5^a^	69.6 ± 13.3
Height (m)	1.76 ± 0.1	1.60 ± 0.1^a^	1.73 ± 0.1
Body mass index (kg/m²)	23.4 ± 3.0	20.8 ± 1.9^b^	23.0 ± 3.0
Body fat (kg)	8.8 ± 5.5	10.2 ± 5.6	9.1 ± 5.6
Body fat (%)	12.2 ± 5.8	21.7 ± 6.1^b^	13.9 ± 6.9
FFM (kg)	64.3 ± 9.1	42.2 ± 5.5^a^	60.3 ±12.1
FFMI (kg/m^2^)	20.6 ± 2.1	16.3 ± 1.5^a^	19.8 ± 2.6
PhA (º)	6.6 ± 0.6	5.5 ± 0.6^a^	6.4 ± 0.7

Data are presented as mean ± SD. FFM, Fat-Free Mass; FFMI, Fat-Free Mass Index; PhA, Phase Angle. Significant difference between sexes (*P* < 0.05), ^a^ using Student *t*-test. ^b^ using Mann-Whitney Test.

Table 2 presents the differences in energy, nutrient and food groups intake according to sex. Male exhibited significantly higher (P < 0.05) total energy (kcal/day) and carbohydrate (g/day) intake and relative protein intake (g/kg per day) than female athletes (P < 0.05). After normalisation for body size, there was no longer a statistical difference in energy and carbohydrate intake between sex. Moreover, male athletes consumed greater amounts of cereals, fruits, meat and eggs, dairy products, and sugar compared to their female counterparts.

**Table 2.** Differences in energy, nutrient, and food group intake according to sex

	Total (*n* = 153)	Male (*n* = 123)	Female (*n* = 30)	P
Energy and nutrients	Mean ± SD	Mean ± SD	Mean ± SD	
Total energy intake (kcal/day)	2696.4 ± 1254	2893.7 ± 612.7	1887 ± 1293.3	<**0.01**
Energy (kcal/kg/day)	39.5 ± 19.2	40.4 ± 13.5	36.1 ± 13.5	0.27
Carbohydrate (g/day)	346.2 ± 205.2	234.9 ± 100.9	374.5 ± 214.9	**<0.01**
Carbohydrate (g/kg/day)	5.1 ± 3.1	5.2 ± 3.3	4.5 ± 2.1	0.51
Carbohydrate (%)	50.6 ± 11.0	49.7 ± 8.1	50.9 ± 11.6	0.46
Protein (g/day)	123.0 ± 70.3	133.3 ± 73.3	81.1 ± 31.7	0.25
Protein (g/kg/day)	1.7 ± 0.9	1.8 ± 1.0	1.5 ± 0.6	**<0.01**
Protein (%)	18.6 ± 6.8	17.6 ± 4.6	18.9 ± 7.2	0.22
Lipid (g/day)	87.0 ± 47.9	91.6 ± 50.7	68.1 ± 27.3	0.16
Lipid (g/kg/day)	1.3 ± 0.7	1.3 ± 0.7	1.3 ± 0.6	0.09
Lipid (%)	30.2 ± 9.2	32.7 ± 7.3	29.2 ± 9.5	0.11
Food groups (portions/day)				
Cereals	6.0 ± 3.8	6.4 ± 4.1	4.4 ± 1.8	**<0.01**
Vegetables	1.4 ± 1.3	1.4 ± 1.4	1.0 ± 0.9	0.06
Fruits	5.6 ± 5.5	6.1 ± 3.7	3.7 ± 3.4	**<0.01**
Beans and nuts	1.7 ± 1.5	1.2 ± 1.1	1.8 ± 1.6	0.09
Meat and eggs	3.8 ± 2.2	4.1 ± 2.3	2.8 ± 1.5	**<0.01**
Dairy products	1.6 ±1.9	1.7 ± 2.0	1.2 ± 0.9	**0.04**
Oil	1.3 ± 1.5	1.4 ± 1.6	1.0 ± 1.2	0.10
Sugar	3.8 ± 5.5	4.2 ± 6.0	2.3 ± 2.2	**<0.01**

Data are presented as mean ± SD. Comparison was performed using Mann-Whitney Test.

The results of the stepwise multiple regression analysis (Table 3) indicated that in Model 1, both lipid (g) and protein (g) intake were associated with phase angle (PhA) in athletes. However, in Model 4, when adjusted for fat-free mass, lipids were no longer significant (P = 0.09). In Model 5, adjusted for age, sex, fat-free mass, and total energy intake, protein intake remained a significant predictor, with the model explaining 47% of the variation in PhA among athletes (R² = 0.47, β = 0.25, P = 0.02).

**Table 3.** Stepwise multiple regression analysis between macronutrient and food groups intake as independent variables in explaining phase angle (PhA) in athletes of different sports (n = 153)

Nutrients	Model 1			Model 2			Model 3			Model 4			Model 5		
	*β*	*P*	95% CI	*β*	*P*	95% CI	*β*	*P*	95% CI	*β*	*P*	95% CI	*β*	*P*	95% CI
Protein (g)	0.42	<0.01*	0.20; 0.51	0.32	<0.01*	0.20; 0.53	0.33	<0.01*	0.15; 0.41	0.25	<0.01*	0.11; 0.32	0.25	0.02*	0.11; 0.33
Lipid (g)	–0.27	<0.01*	–0.50; –0.10	–0.40	<0.01*	0.02; 0.05	–0.17	0.03*	–0.35; –0.02	–		–	–		
Adjusted R²	0.12			0.21			0.37			0.46			0.47		

Food groups	Model 1			Model 2			Model 3			Model 4			Model 5		
	*β*	*P*	95% CI	*β*	*P*	95% CI	*β*	*P*	95% CI	*β*	*P*	95% CI	*β*	*P*	95% CI
Fruits	0.16	0.03*	0.02; 0.04		–			–			–			–	–
Meat & eggs	0.33	<0.01*	0.06; 0.17	0.38	<0.01*	0.09; 0,19	0.29	<0.01*	0.05; 0.15	0.25	<0.01*	0.01; 0.04	0.26	<0.01*	0.04; 0.13
Adjusted R²	0.13			0.25			0.37			0.46			0.46		

*P ≤ 0.05. β = Standardised Coefficients. Model 1: unadjusted. Model 2: Adjusted for age. Model 3: Adjusted for age and sex. Model 4: Adjusted for age, sex, and fat-free mass (kg). Model 5: Adjusted for age, sex, fat-free mass (kg), and total energy intake (kcal).

Regarding food group consumption (Table 3), the intake of fruits and meat and eggs was associated with PhA in Model 1. However, after adjustments for age, sex, and fat-free mass, only meat and eggs remained a significant predictor of PhA. This effect persisted even after adjusting for total energy intake in Model 5, explaining 46% of the variance in PhA (R² = 0.46, β = 0.26, P = 0.01).

**Figure 1.**
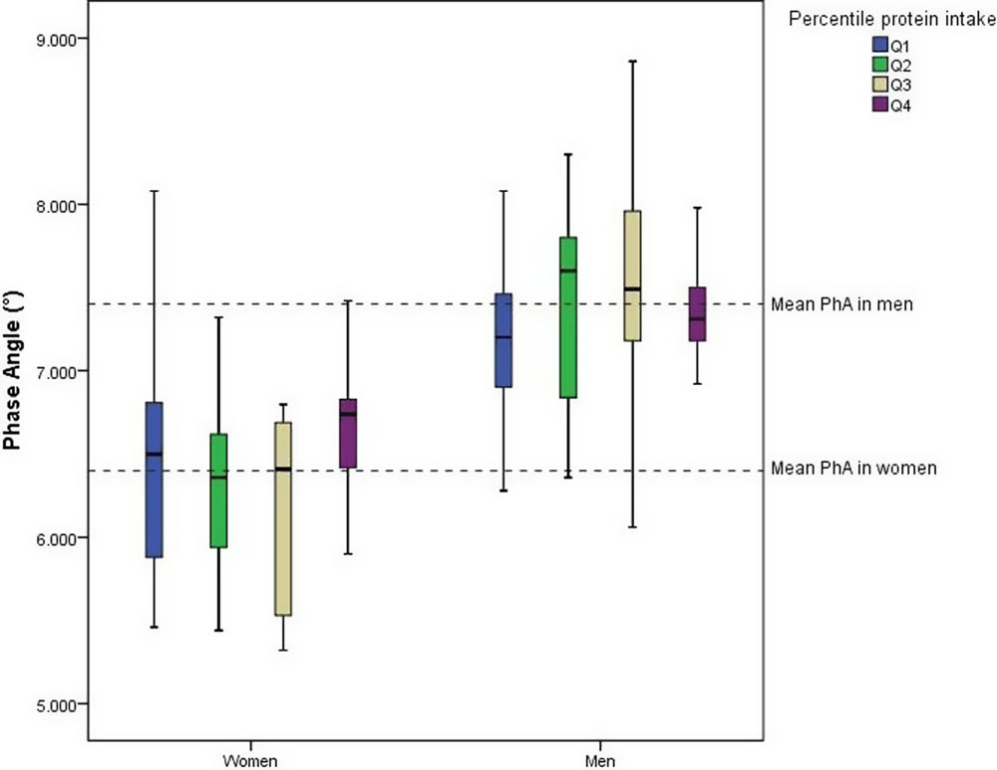
Distribution of Phase Angle (°) by sex and quartile of protein intake in athletes.

Athletes were divided into quartiles (Q1–Q4) of protein intake (g/kg per day) for males and females. For females, the cut-off points were: Q1 (≤1.21 g/kg per d), Q2 (1.21–1.40 g/kg per d), Q3 (1.41–1.83 g/kg per d), and Q4 (>1.83 g/kg per d For males, the cut-offs were: Q1 (≤1.25 g/kg per d), Q2 (1.25–1.61 g/kg per d), Q3 (1.62–2.14 g/kg per d), and Q4 (>2.14 g/kg per d). Figure 1 illustrates the distribution of phase angle (PhA) in athletes of both sexes across quartiles of protein intake. Regardless of the quartile, male athletes demonstrated higher mean PhA values compared to female athletes, as highlighted by the dashed lines representing the mean PhA for each sex. The highest mean PhA was observed in the highest quartile of protein intake in female athletes, whereas male athletes exhibited higher mean PhA values in Q2 and Q3 in relation to Q1 and Q4. The interquartile ranges (boxes) highlight greater variability in PhA among male athletes, particularly in Q3 and Q4, compared to female athletes.

## Discussion

To the best of our knowledge, this is the first study investigating the relationship between PhA and dietary intake in athletes. The key finding of this study is that protein intake is a predictor of PhA, independent of variables such as sex, age, and fat-free mass. Although some studies with different populations have provided results on the influence of dietary intake on PhA,^(13,28,29)^ our study’s focus on athletes provide unique insights.

It is important to highlight that the literature on the associations between diet and bioimpedance parameters is both scarce and often contradictory. This inconsistency may be attributed to the different technologies and frequencies used in bioimpedance devices across studies. Therefore, comparisons between studies should be interpreted with caution, and we have presented the specific characteristics of the devices used in each study. De França *et al.* ^(28)^ showed that both dietary quality and muscle mass were protective factors against low PhA in Brazilian adults. The PhA was calculated using Xc and R values obtained from a whole-body tetrapolar bioimpedance (Biodynamics, Model 450, USA), applying an alternating electric current of 800 μA and 50 kHz). In contrast to our study, the authors observed that only the overall diet, rather than individual nutrients or food groups, was associated with PhA, suggesting the importance of a combination of quantitative and qualitative dietary parameters.^(28)^

In a cross-sectional observational study with adults, adherence to the Mediterranean diet showed a direct and independent association with PhA in male and female participants (13). Additionally, in the multivariate analysis, adherence to this diet was the major determinant of PhA compared to age and body weight. In their study, the PhA was calculated using Xc and R values obtained from the tetrapolar BIA 101 RJL equipment (Akern Bioresearch, Italy) using an 800-µA current at a single frequency of 50 kHz.

Given that PhA measures the cell’s capacity to act as a capacitor,^(2)^ a strong association with fat-free mass is anticipated. Adult athletes, characterised by a high content of fat-free mass and consequently greater muscle cellularity (indicative of higher Xc) and body water content (indicative of lower R), are expected to present higher PhA compared to their non-athletic counterparts.^(13,14)^ This suggests superior cell function in the athletic group.^(10)^ Marra *et al.* ^(14)^ corroborated this by showing that fat-free mass was the most determinant of PhA in a multivariate regression model.

In our study, male athletes had higher fat-free mass, FFMI, and relative protein intake (g/kg per day) compared to female athletes, potentially explaining the higher PhA in men. Additionally, protein intake was found to be an independent predictor of PhA. Optimal protein intake is essential for maintaining muscle integrity, growth, and recovery in athletes, preventing protein catabolism and muscle wasting during the training season.^(30)^ As muscle is primarily composed of water and amino acids, a higher PhA is likely in athletes with greater muscle mass consuming a high-protein diet.

It is noteworthy that both male and female athletes in our study exhibited a mean protein consumption in accordance with nutritional guidelines (i.e. >1.2 g/kg per d) (Table 2)^(30)^ although the distribution by quartiles (Fig. 1) reveals that athletes in Q1 consume protein close to or lower than this recommended amount (i.e. Q1 for female: ≤ 1.21 g/kg per d and Q1 for male athletes: ≤ 1.25 g/kg per d). Our findings support the positive association between protein intake and PhA, where female athletes exhibited higher mean PhA values in Q4 than in all previous quartiles, while male athletes exhibited higher mean PhA values in the Q2 and Q3 than those in the first quartile. Our results suggest the importance of adequate protein intake (>1.2 g/kg per d), especially in situations that could decrease PhA, such as injuries or weight loss diets.

Interestingly, the protein intake in Q4 of female athletes was >1.83 g/kg per d, and inside the range ingested by men in Q3 (i.e. 1.62–2.14 g/kg per d). suggesting that a daily protein intake into this range is preferably for a higher PhA in athletes. Protein consumption, in parallel with physical training, stimulates pathways that increase protein synthesis and decrease protein degradation, favouring muscle repair and intracellular water accumulation,^(31,32)^ potentially resulting in higher PhA values. However, protein intake above the upper recommended intake of 2.2 g/kg per d for the athletes in ‘normal circumstances’ seems not to further contribute to gains in muscle mass.^(30)^

In a meta-analysis, Morton and colleagues^(33)^ showed that FFM did not increase beyond total protein intakes of aprox. 1.6 g/kg per d. Given that the confidence interval of the studies’ estimates varied from 1.03 to 2.20 and the potential differences between athletes in protein needs, the authors suggested the intake of aprox. 2.2 g protein/kg per d for those seeking to maximise resistance training-induced gains in FFM. We infer that it is also true for PhA as in our study, the PhA of male athletes in Q4 was below the mean PhA, even with a protein intake > 2.14 g/kg per d. This could be an indication that, while not solely reliant on muscle mass, the relationship between protein and PhA may be limited where the excess of protein intake doesn’t automatically increase it, especially if at the expense of dietary calories, water or other nutrients critical for cellular health (e.g. omega3 fatty acids).

Interestingly, consistent with previous research,^(20,29)^ we identified a positive association between PhA and meat and eggs intake, regardless of sex, age, fat-free mass, and energy intake indicating that the quality of protein sources may have an additional impact beyond protein intake. A meta-analysis of randomised controlled trials carried out by Lim *et al.* ^(34)^ compared the effect of animal (i.e. whey, casein, milk protein, dairy products, and beef) vs plant protein sources (i.e. soy, pea protein, and rice) with or without resistance training on fat-free mass and muscle strength in adults. The findings indicated that both protein sources supported an increase in absolute and percentage fat-free mass. Although there was a slight favouring effect of animal protein on percentage fat-free mass (∼0.50% higher), no significant difference was observed when analysed according to resistance training.^(34)^ Unfortunately, the authors did not assess PhA, so it is not possible to establish a direct association. The lack of studies on different protein sources and their association with PhA warrants further investigation, especially in athletes. Meanwhile, it is important to mention that the consumption of meat and eggs was high among our study group, with an average of 3.8 portions/d. These foods were the main sources of proteins, especially high biological value proteins in their diets, which may have a direct influence on muscle mass due to their higher bioavailability of essential amino acids, explaining their positive effect on PhA.

This is the first study to investigate associations between quantitative (macronutrient intake) and qualitative (groups of food) aspects of the diet and PhA in athletes. However, some limitations should be acknowledged. Like all cross-sectional studies, our study did not allow us to identify a causal association between dietary intake and PhA or to determine the amount of food and nutrients needed to improve PhA in athletes. Our findings indicate that while protein intake influences PhA in athletes, this effect may not apply to non-athletic adults. The exact mechanism behind this association remains unclear, and longitudinal studies are needed to address this and overcome the limitations of our cross-sectional design. Additionally, given the young age of our participants, the results may not extend to older populations. Future research should explore how aging impacts the relationship between protein intake and bioelectrical impedance parameters outcomes, including PhA. A further limitation concerns the inherent errors in food consumption assessment within nutrition epidemiology. While there is currently no gold standard for measuring energy intake in athletes, the 24-hour dietary recall method is recognised as appropriate for the general population and is usually applied similarly to athlete groups.^(35,36)^ Although this method has known weaknesses, the use of trained researchers, as employed in our study, decreases the risk of bias and improves the quality of the measure.^(22)^ Furthermore, we recognise that certain supplements can impact fat-free mass composition and fluid compartmentalisation, potentially influencing PhA. We did not perform any type of analysis to quantify the effect of supplement intake on the PhA. However, all nutrients ingested through supplements were accounted for based on their biochemical classification (e.g. amino acids were included in protein intake), whenever possible. We expect that the influence of supplements on body composition is captured through the inclusion of the fat-free mass variable in our regression model. The small number of female athletes also restricts the generalizability of the results. Further research with larger and specific athlete populations is necessary to establish adequate nutrient amounts for achieving the best PhA values.

The findings of this study may benefit healthcare professionals by enabling better monitoring of their patients’ progress from a nutritional perspective. However, the results obtained by bioimpedance cannot be directly compared between different devices, as the varying technologies used can introduce significant biases. Therefore, it is crucial that the equipment is always properly calibrated and that results are compared using the same device and standardised measurement procedures.

## Conclusion

The positive association between protein intake and PhA observed in this cross-sectional study highlights the potential of this approach for monitoring nutrition changes in athletes. However, additional research is needed to validate these findings and to identify further nutritional interventions that would enhance athletes’ PhA.
